# Radioprotective Role of Peroxiredoxin 6

**DOI:** 10.3390/antiox8010015

**Published:** 2019-01-05

**Authors:** Mars G. Sharapov, Vladimir I. Novoselov, Sergey V. Gudkov

**Affiliations:** 1 Laboratory of Mechanisms of Reception, Institute of Cell Biophysics of the Russian Academy of Sciences, 142290 Pushchino, Russia; novoselov-vi@rambler.ru; 2 Wave Research Center, Prokhorov General Physics Institute of the Russian Academy of Sciences, 119991 Moscow, Russia; s_makariy@rambler.ru; 3 Department of Experimental Clinical Studies, Moscow Regional Research and Clinical Institute (MONIKI), 129110 Moscow, Russia; 4 The Institute of Biology and Biomedicine, Lobachevsky State University of Nizhni Novgorod, 603950 Nizhni Novgorod, Russia

**Keywords:** peroxiredoxin 6, ionizing radiation, radioprotection, antioxidant activity

## Abstract

Peroxiredoxin 6 (Prdx6) is a member of an evolutionary ancient family of peroxidase enzymes with diverse functions in the cell. Prdx6 is an important enzymatic antioxidant. It reduces a wide range of peroxide substrates in the cell, thus playing a leading role in the maintenance of the redox homeostasis in mammalian cells. Beside peroxidase activity, Prdx6 has been shown to possess an activity of phospholipase A2, an enzyme playing an important role in membrane phospholipid metabolism. Moreover, Prdx6 takes part in intercellular and intracellular signal transduction due to its peroxidase and phospholipase activity, thus facilitating the initiation of regenerative processes in the cell, suppression of apoptosis, and activation of cell proliferation. Being an effective and important antioxidant enzyme, Prdx6 plays an essential role in neutralizing oxidative stress caused by various factors, including action of ionizing radiation. Endogenous Prdx6 has been shown to possess a significant radioprotective potential in cellular and animal models. Moreover, intravenous infusion of recombinant Prdx6 to animals before irradiation at lethal or sublethal doses has shown its high radioprotective effect. Exogenous Prdx6 effectively alleviates the severeness of radiation lesions, providing normalization of the functional state of radiosensitive organs and tissues, and leads to a significant elevation of the survival rate of animals. Prdx6 can be considered as a potent and promising radioprotective agent for reducing the pathological effect of ionizing radiation on mammalian organisms. The radioprotective properties and mechanisms of radioprotective action of Prdx6 are discussed in the current review.

## 1. Introduction

**Figure 1:**
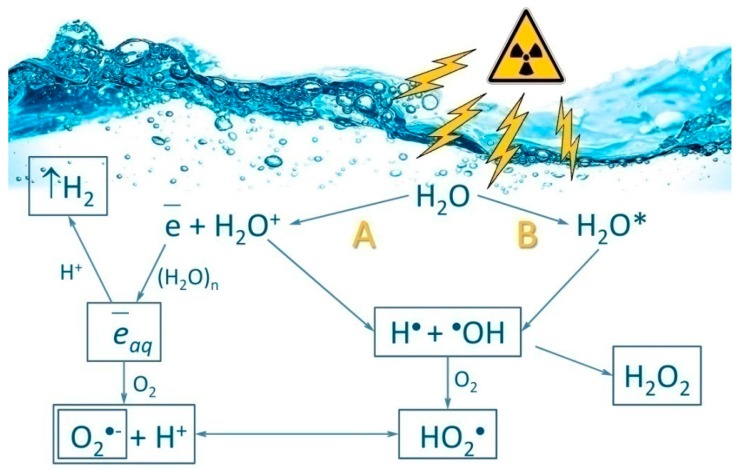
Schematic representation of water radiolysis. The reactions are ionization (**A**) and excitation (**B**), which produce reactive oxygen species (ROS). Primary (HO^•^, H^•^) and secondary (H_2_O_2_, O_2_^•−^, HO_2_^•^) ROS involved in oxidative stress are produced by ionizing radiation. e¯_aq—_solvated electron; H_2_O*—excited water molecule.

Ionizing radiation is the flow of photons, elementary particles, and nuclear fission fragments capable of ionizing matter. The ionization process is usually defined as the conversion of neutral atoms or molecules into ions and radicals [1]. Ionizing radiation has a direct and indirect influence on living organisms. The direct effect includes damaging biological molecules via immediate contact with a quantum or particle of ionizing radiation. Indirect influence is related to the formation of water radiolysis products in the cell, namely reactive oxygen species (ROS) (Figure 1). The most widely known members of the ROS group are the superoxide anion radical (O_2_^•−^), hydroperoxide radical (HO_2_^•^), hydrogen peroxide (H_2_O_2_), hydroxyl radical (HO^•^), singlet oxygen (^1^O_2_), etc. [2]. Every 100 electron-volts of absorbed ionizing radiation energy generates on average 2.4 HO^•^ radicals, 2.8 solvated electrons, 0.4 hydrogen atoms, 0.8 H_2_O_2_ molecules, 0.4 H_2_ molecules and much less of other compounds [3].

**Figure 2:**
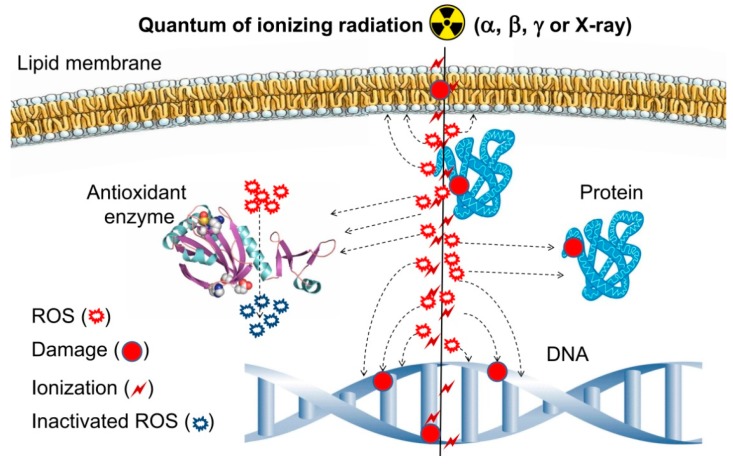
Schematic representation of the effects of ionizing radiation on a living cell.

The situation when intracellular ROS concentration exceeds the capacity of antioxidant systems is called oxidative stress. Oxidative stress is accompanied by processes dangerous for cell living and functioning, such as lipid peroxidation [4], protein oxidation [5], and nucleic acid modification [6]. Oxidative DNA damage is closely related to mutagenesis and carcinogenesis processes and development of severe diseases [7,8,9]. The damage of DNA molecules is also one of the basic reasons for post-exposure death of living systems. A significant portion (around 70–80%) of DNA damage caused by radiation is due to ROS formed during water radiolysis, and only 20%–30% is caused by direct absorption of high-energy quanta of ionizing radiation by target molecules [10,11,12]. (Figure 2).

Living organisms have developed different protective mechanisms in the course of evolution, the leading role among them belonging to antioxidants, because they are the suppressors of free radical oxidation alleviating the consequences of exposure to ionizing radiation [12,13]. Due to antioxidants, living systems are capable of maintaining the physiological ROS level and, as a result, their redox homeostasis. The antioxidant protection system includes low molecular weight compounds and antioxidant enzymes [14,15,16]. Low molecular weight compounds include vitamins, bioflavonoids, antioxidant hormones, low molecular weight thiols, etc. The most important antioxidant enzymes are superoxide dismutases (SODs), catalase, glutathione peroxidases (GPxs), glutathione reductases (GRs), thioredoxins (Trxs), and peroxiredoxins (Prdxs). A significant interest in these enzymes is caused by the fact that peroxiredoxins is an evolutionarily ancient family of peroxidases. Prdxs are detected in all living organisms, both in aerobic and in anaerobic ones. During the increase of organization complexity from bacteria and protists to multicellular organisms, the increase of the number of isoforms of peroxiredoxins took place. Hence, three peroxiredoxin isoforms have been detected in bacteria [17], five in yeast [18], six in mammals [19], and nine in plants [20]. The increase of Prdx isoform number in eukaryotes is caused by specialization of the enzymes in intracellular compartments and by their substrate specificity [21]. The evolutionary success of peroxiredoxins, which allowed them to spread across the whole living world, is due to the following reasons: (1) wide substrate specificity; (2) catalytic efficiency; (3) multifunctionality; (4) their involvement in different cellular processes.

**Figure 3:**
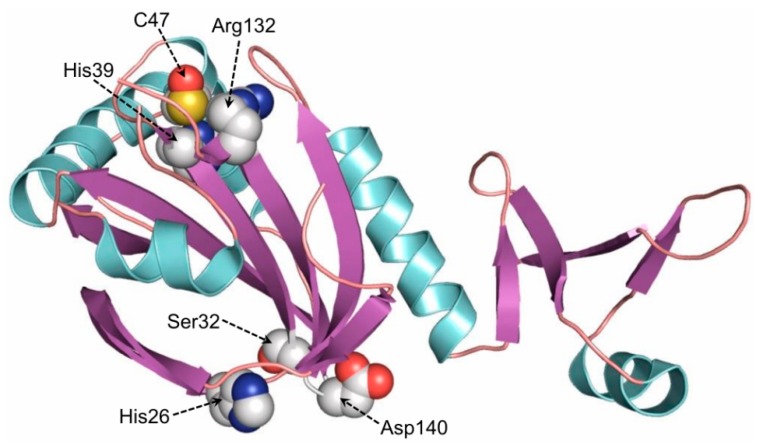
The schematic structure of human Prdx6 (Peroxiredoxin 6). Amino acid residues in the peroxidase catalytic center (His39, Cys47, Arg132) and phospholipase A2 active center (His26, Ser32, Asp140) are shown. The structure was built in Pymol.0.99.

Prdxs perform their catalytic function by a conservative cysteine residue (C_P_—peroxidatic cysteine) in the active site, and they do not contain any auxiliary cofactors [16,22]. Prdxs play an important role in the regulation of ROS level in the cell, because they are capable of reducing various inorganic and organic peroxides [23,24,25,26,27,28,29,30]. Among mammalian peroxiredoxins (Prdx1–6), Prdx6 draws particular attention because of its ability to reduce the widest range of substrates, including H_2_O_2_, peroxynitrite, alkyl peroxides, phospholipid peroxides, etc. [31,32,33]. Due to its antioxidant properties, Prdx6 plays an important role in maintaining the redox balance in mammalian cells [34,35]. Animals with *PRDX6* gene knockout, despite normal expression of the genes encoding other antioxidant enzymes, display a high sensitivity to oxidative stress (caused by hyperoxygenation, effect of peroxides, paraquat, etc.), which is accompanied by an elevated level of oxidative damage of animal organs and tissues [30]. Beside peroxidase activity, Prdx6 has been shown to possess an activity of Ca^2+^-independent phospholipase A2 (aiPLA2), which is normally expressed only under acidic conditions (in lysosomes and lamellar bodies, at pH 4–5) and plays an important role in the metabolism of phospholipids and intracellular/intercellular signal transduction [36,37]. Thus, Prdx6 is a unique bifunctional enzyme (Figure 3) participating in many cellular processes [38].

This publication is part of a Forum on “Peroxiredoxin 6 as a Unique Member of the Peroxiredoxin Family”. The radioprotective role of Prdx6 in mammalian organism and possible mechanisms of its radioprotective effect are discussed in the present review.

## 2. Regulation of *PRDX6* Expression

The character of expression of different peroxiredoxin isoforms in mammals exhibits cellular, tissue and organ specificity. The major factor affecting the level of *PRDX1*–*6* gene expression is elevation of the ROS level, which can be caused by external and internal factors. It has been demonstrated that the action of hyperoxygenation, pro-oxidants (heme, transition metals, xenobiotics), hydroperoxides (of organic and inorganic nature), UV and ionizing radiation leads to an elevation of *PRDX1*–*6* expression level [39,40,41,42,43,44]. The major role in the regulation of *PRDX1*–*6* gene expression belongs to transcription factor NRF2 [45,46,47,48]. Along with NRF2, other transcription factors also participate in *PRDX1*–*6* gene expression, such as HIF, AP-1, NF-kB, c-Myc, C/EBP, FOXO3, etc. [49,50,51,52,53,54,55].

**Figure 4:**
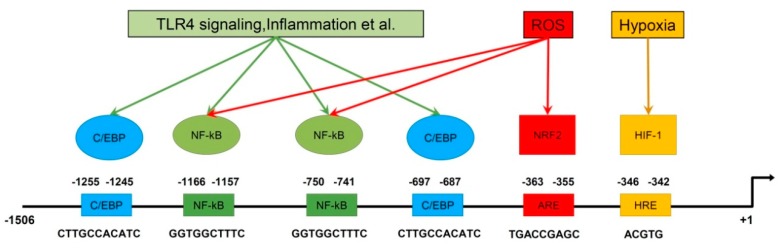
Schematic representation of the regulation of *PRDX6* expression. The *PRDX6* promoter and binding sites of different transcription factors are shown.

It is worth mentioning that *PRDX6* expression is regulated by numerous transcription factors (Figure 4). Factors NRF2, HIF1α and C/EBPβ enhance *PRDX6* expression, while NF-kB has a suppressive effect on the expression level of PRDX6. Analysis of the *PRDX6* gene promoter showed the presence of binding sites for each of the aforementioned transcription factors [56,57].

Beside transcription factors, other enzymes, immunomodulators, etc. are also involved in the regulation of *PRDX1*–*6* expression [39,50,58,59,60]. It has been shown recently, that nucleophosmin (NPM1), a DNA/RNA chaperone, stimulates *PRDX6* expression, and NPM1 gene knockdown or addition of a specific inhibitor of nucleophosmin, NSC348884, to cell cultures suppresses *PRDX6* expression. On the contrary, an increase of NPM1 level also provides an increase of Prdx6 level [61]. Another important mechanism of peroxiredoxin gene expression regulation is mediated by microRNAs [62,63,64]. *PRDX6* expression is suppressed via miR-24-3p, which specifically binds to the 3′-untranslated region of mRNA, thus suppressing *PRDX6* gene expression [65]. The miR-24-3p level in gastric cancer cell line N87 is significantly lowered, which, in turn, stimulates cancer cell growth and metastasis formation [65].

Thus, *PRDX6* gene expression level can be regulated by a complex of factors, which allows «flexible» reaction of the transcriptional machinery on the changing of internal and external conditions for the cell, accompanied by alteration of ROS level.

## 3. Role of Endogenous Prdxs in Radioresistance of Mammalian Cells

Adaptive induction of Prdxs synthesis occurs in cells in response to exposure to ionizing radiation and other factors that provoke an elevation of cellular ROS level. High radioprotective potential of peroxiredoxins has been shown in a series of experiments in animal models and cell cultures. UV and X-ray irradiation of rat skin has been shown to increase Prdx1, Prdx2, Prdx3 and Prdx6 expression level [43,66], and X-ray irradiation of murine testes has been testified to lead to a multifold increase of Prdx1 and Prdx2 [44]. Besides that, exposed mice have displayed a significant increase in Prdx1 and Prdx2 expression levels in the brain [67,68,69], and in Prdx6 expression level in the liver and spleen [70,71].

Many ionizing radiation-resistant cancer cell lines demonstrate high-level peroxiredoxin expression. For instance, a leading role of Prdx2 has been shown in the radiosensitivity of human colon cancer cells (HCT116, Caco-2, T84 and LoVo) and breast cancer cells (MCF + FIR3) [72,73,74]. Suppression of *PRDX2* expression significantly weakens the resistance of these cancer cells to radiation. Aggressive radioresistant brain tumor species, glioblastoma, shows a high *PRDX4* expression level [75]. *PRDX4* gene knockdown in glioblastoma cells leads to increased sensitivity of the cells to ionizing radiation, suppression of growth, and metastasis formation [76]. A notable elevation of Prdx6 level has been detected in many cancer species, many of which have high radioresistance (Table 1). 

Table 1 show that antioxidant function of Prx6 plays the most crucial role in radioresistance of cancer cells, but beside that, it’s signaling and regulatory function is also important, which will be discussed below. In vitro and in vivo experiments showed that suppression of *PRDXs* genes expression in cancer cells led to loss of their radioresistance, which allows to consider them as potential targets during cancer radiotherapy [90,91,92,93,94].

## 4. Application of Exogenous Prdx6 as a Radioprotector

The level of ROS formation in many cases of exposure to ionizing radiation exceeds the ability of living cells to eliminate them, and massive damage of nucleic acids, proteins, and lipids is observed in such conditions. Damage of biological macromolecules is one of the main reasons of post-irradiation death of animals [95]. Radioprotective drugs are used in practice to prevent the harmful consequences of the action of ionizing radiation. On the whole, radioprotective agents can be divided into two classes: (1) compounds preventing damage of macromolecules; (2) compounds stimulating post-irradiation recovery. Low-molecular antioxidants, enzymatic antioxidants, antioxidant synthesis inducers, molecule stabilizers, and compounds causing hypoxia can be assigned to the first class. A large number of compounds affecting post-irradiation recovery of macromolecules and anti-apoptotic agents, compounds involved in chemical reparation, etc. can be assigned to the second class [96,97,98]. Basically, radioprotective compounds differ in the structure and mechanism of action. The main groups of radioprotective compounds are listed in Table 2 by structure and mechanism of action. More detailed data on the main classes of radioprotective compounds can be found in reviews [96,99].

It should be noted that actively dividing cells (epithelial, embryonic and stem cells) are the most sensitive to the action of ionizing radiation, that is why this type of cells needs particular radioprotection. Development of novel effective radioprotective compounds acting primarily by preserving these tissue types is an important and actual task. In this regard, the promising direction in the creation of effective radioprotective agents is the application of antioxidant enzymes, which are several orders of magnitude more active than the currently used low-molecular compounds. Attempts to use such enzymes as catalase and superoxide dismutase were made earlier, but they have all been unsuccessful [100,101,102]. This is explained first of all by the fact that organic hydroperoxides (products of oxidation of proteins, lipids, etc.) comprise a large portion of the whole ROS pool produced as a result of irradiation, while catalase and superoxide dismutase are not capable of neutralizing such types of ROS. As mentioned before, the peroxiredoxin family is the group neutralizing the widest range of ROS, which is why the application of Prdxs as radioprotectors seems the most promising approach.

Application of exogenous recombinant Prdx6 in model animal experiments has shown high efficiency in the treatment of disorders attended by oxidative stress, such as severe chemical and thermal burns of the upper respiratory tract, acute inflammation of respiratory organs caused by bacterial endotoxins (LPS), and ischemia-reperfusion injuries [103,104,105,106,107]. Infusion of Prdx6 before the action of the aforementioned factors provides suppression of oxidative stress, thus preventing the damage of actively proliferating tissues. Prdx6 infusion after the damage, for example, after thermal burns of the upper respiratory tract, leads to a faster recovery of tissues, suppression of cell necrosis, and apoptosis [108,109]. Thus, the obtained experimental data has evidenced that exogenous Prdx6 possesses a high antioxidant potential and could be promising in the treatment of pathologies accompanied by the development of oxidative stress, particularly, those caused by ionizing radiation.

**Figure 5:**
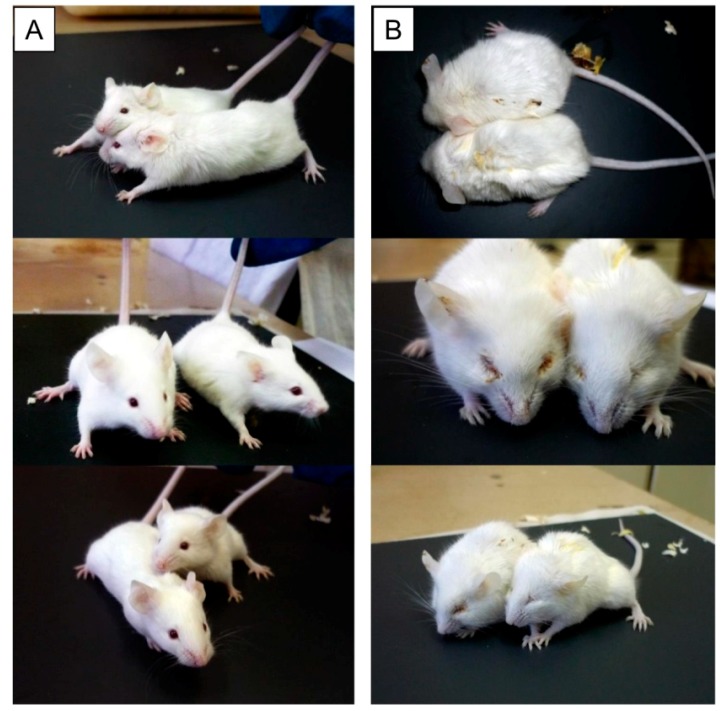
Mice after X-ray irradiation at a dose of 7 Gy (after 7 days). (**A**) Intravenous administration of Prdx6 before irradiation (20 µg/g), (**B**) administration of 0.9% NaCl in the same volume. Irradiation of animals was carried out on RUT-15 (Moscow, Russia) at a dose rate of 1 Gy per minute.

The survival rate of 6-week male mice (line Kv:SHK) was studied after irradiation with sublethal and lethal doses (5–11 Gy) and intravenous infusion of recombinant Prdx6 solution before X-ray action [110,111]. The radioprotective effect of exogenous Prdx6 was maximal upon intravenous injection 15–30 min before irradiation. The optimal concentration was about 20 µg/g of body weight. Injection of a larger amount of the protein did not lead to significant growth of its radioprotective effect. The radioprotective effect of Prdx6 was clearly visible 7 days after irradiation. The control group which had not received Prdx6 injection before irradiation demonstrated typical symptoms of acute radiation syndrome, whereas animals which received an intravenous injection of Prdx6 at that moment did not differ from intact, non-irradiated animals (Figure 5).

**Figure 6:**
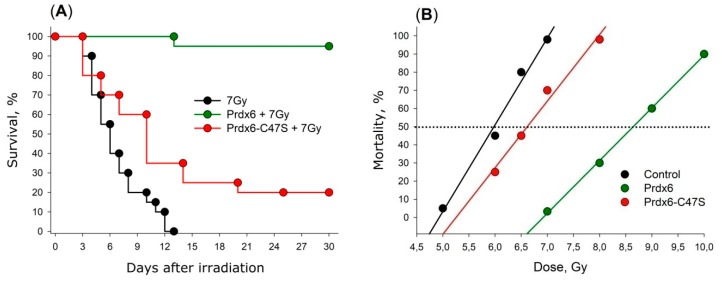
(**A**) Effect of intravenous administration of Prdx6 and Prdx6-C47S (20µg/g) on the survival of mice exposed to X-rays at a lethal dose of 7 Gy. (**B**) Effect of intravenous administration of Prdx6 and Prdx6-C47S (20µg/g) on the mortality of mice at sublethal and lethal doses of X-rays.

Mutant variant Prdx6-C47S, which has no peroxidase activity, was studied in the same manner. It was surprising that even this variant had a slight radioprotective effect. The results on the survival rate of mice receiving Prdx6 and its mutant form Prdx6-C47S are present in Figure 6A. An average lifespan of irradiated mice was 7 days, and the maximal survival time was 13 days. Prdx6 injection 15 min before X-ray irradiation significantly increased the survival of animals (*p* < 0.001). Approximately 95% of animals were alive over 30 days. The Figure 6B shows linear dose dependencies of 30-day survival of mice [12]. Dose reduction factor (DRF) for Prdx6 was around 1.4 [110,111].

Table 2 lists DRF values for some of the radioprotective compounds. As it can be seen from Table 2, there are compounds with higher DRF values, moreover, there is a large number of compounds with comparable DRF values. However, the noticeable competitive advantage of Prdx6 over other preparations listed in Table 2 is lack of toxicity and adverse effects [112]. For example, for KGF and G-CSF, course application is supposed to be able to stimulate tumor growth [113]. Injection of Interleukin-1 often causes significant temperature elevation, vomiting, headache, and fatigue [114]. Sulfhydryl preparations are effective as radioprotectors in doses close to acute toxic doses [98]. 

**Figure 7:**
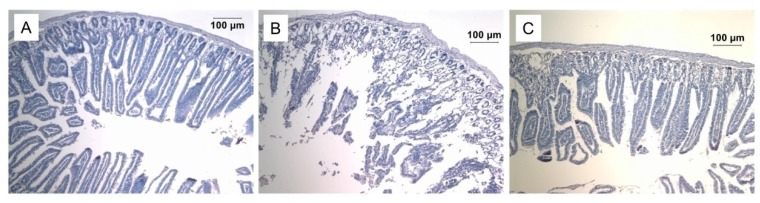
Effect of intravenous administration of Prdx6 (20µg/g) 15 min before exposure to X-ray radiation (dose 10 Gy) on the small intestine morphology. Micrographs of the small intestine stained with hematoxylin-eosin (×100) of (**A**) intact mice, (**B**) irradiated mice, (**C**) irradiated mice that received Prdx6.

Death after exposure to doses of ionizing radiation close to 10 Gy is usually caused by injuries of the digestive tract, particularly the small intestine. We found that injection of Prdx6 significantly protects the small intestine from the damaging effects of ionizing radiation (Figure 7). We must note that the protective effect of exogenous Prdx6 has also been shown earlier on an ischemia–reperfusion model of small intestine injury [106,107].

**Figure 8:**
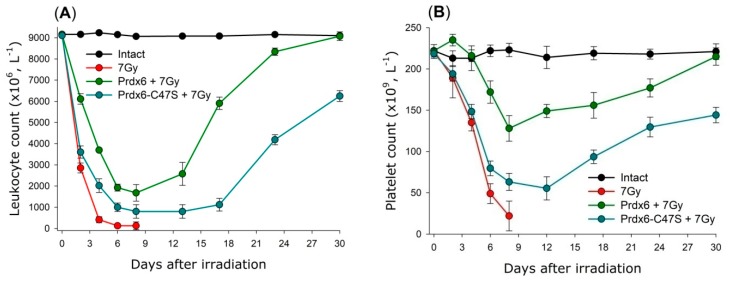
Effect of intravenous injection of Prdx6 (20µg/g) 15min before X-ray irradiation (dose 7 Gy) on the leukocyte (**A**) and platelet (**B**) count in the peripheral blood of irradiated mice. The data are given as means ± SEM (*n* = 5).

It is known that the death of small laboratory animals (mice and rats) after exposure to ionizing radiation at doses of 3–10 Gy is due to the hematopoietic syndrome as a result of mass death of bone marrow cells [115]. This is also accompanied by a depletion of the bone marrow stem cell depot. As a result, the amount of blood cells in peripheral blood changes, causing immune deficiency and development of haemorrhagia. Intravenous injection of Prdx6 before irradiation significantly decreases the severity of radiation-induced leukopenia and thrombocytopenia (Figure 8) [110,111].

**Figure 9:**
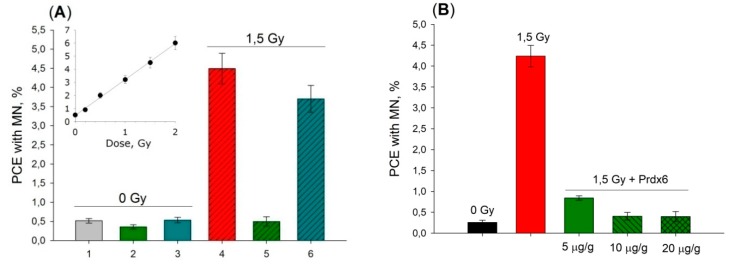
Effect of intravenous injection of Prdx6 and Prdx6-C47S 15 min before irradiation with X-rays (dose 1.5 Gy) on the formation of PCE with MN in the bone marrow cells of mice. The data are given as means ± SEM (*n* = 5). (**A**) 1—intact mice; 2—non-irradiated mice received Prdx6 (20 µg/g); 3—non-irradiated mice received Prdx6-C47S (20 µg/g); 4—irradiated mice; 5—irradiated mice received Prdx6 (20 µg/g), 6—irradiated mice received Prdx6-C47S (20 µg/g). (**B**) Dose-dependent reduction of PCE with MN level depending on intravenous Prdx6 (5, 10, 20 µg/g) injection 15 min before 1,5 Gy irradiation was shown.

Besides that, Prdx6 injection was shown to suppress genomic DNA damage significantly in bone marrow cells under action of X-ray radiation at a dose of 1.5 Gy. Linear dose dependence of micronuclei (MN) formation in polychromatophylic erythrocytes (PCE) after total X-ray irradiation of mice (Figure 9) showed that injection of Prdx6 in 20 µg/g dose 15 min before irradiation by 1.5 Gy dose led to DNA damage compared to that observed in mice irradiated by merely 0.1–0.2 Gy. It is interesting to note that the mutant variant, Prdx6-C47S, did also demonstrate genoprotective properties (Figure 9), which obviously cannot be related to antioxidant properties of this protein.

Apparently, the radioprotective effect of Prdx6 is mediated by several components. First of all, by the peroxidase activity of Prdx6, which provides neutralization of a wide range of peroxide substrates, including long-lived reactive protein species (LRPS), Prdx6 eliminating LRPS more effectively than it was shown earlier for such compounds as inosine, guanosine, vitamin C, and l-methionine [116,117]. Secondly, it is caused also by the signaling and regulatory function of Prdx6, which facilitates triggering of recovery processes in stress conditions and is not related to its peroxidase activity [110,111].

## 5. Molecular Mechanisms of Radioprotective Effect of Endogenous and Exogenous Prdx6

**Figure 10:**
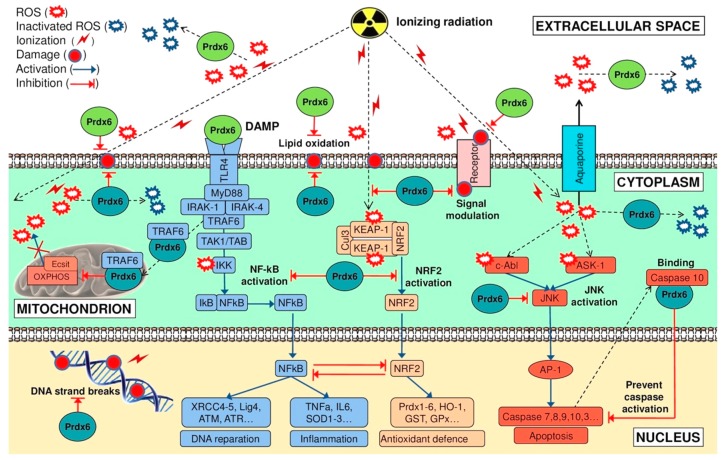
Schematic representation of the molecular mechanism of radioprotection by endogenous (cyan) and exogenous (green) Prdx6 under the action of ionizing radiation.

There is no doubt that the most important component of the radioprotective effect of exogenous Prdx6 is its peroxidase activity, which is proved by a significant decrease of the radioprotective effect after injection of mutant Prdx6-C47S variant lacking peroxidase activity (Figure 6). We noticed earlier that Prdx6 neutralizes the widest range of hydroperoxides compared to other peroxiredoxin family members. It is known that the action of ionizing radiation results in the generation of ROS of various natures, including peroxides of phospholipids and long-lived reactive protein species (LRPS), which are effectively neutralized by Prdx6. Finally, an important role in the radioprotective action of Prdx6 is played by its signaling and regulatory activity. The proposed scheme of possible events after the action of ionizing radiation on the cell and the role of exogenous and endogenous Prdx6 in these processes is shown in Figure 10. 

### 5.1. Endogenous Prdx6

Prdx6 is an important antioxidant enzyme, which is related to its wide substrate specificity and high catalytic efficiency. However, Prdx6 is important not only as an antioxidant, but also as a signaling and regulatory protein. Prdxs are known to be capable of «fine sensitivity» to alterations in the redox status of the cell due to high lability of thiol groups in the active site. Depending on the ROS level in the cell and the redox degree of C_P_ (peroxidatic cysteine), Prdxs direct cell development via interaction with key regulatory proteins, «switching» the cell from one signaling pathway to another [22]. Due to the slower process of sulfenic acid (C_P_-SOH) reduction in the active site of Prdxs compared to C_P_-SH oxidation kinetics, accumulation of oxidized peroxiredoxins takes place in the cell [118]. In this regard, during reduction of oxidized cysteine C_P_-SOH, peroxiredoxins can form intermolecular disulfide bonds with reducing proteins (Trx1, Trx2, PDI, πGST) and other thiol (–SH) group-containing proteins, such as transcription factors, kinases, phosphatases, receptors, ionic channels, etc., thus modulating their activity and affecting many cellular processes (Figure 10). Particularly, Prdxs localized in the cell nucleus interact with the most important transcription factors: NF-κB, p53, C-Myc, PTEN, p53, etc., thus indirectly affecting expression of many genes [22,119]. 

Excessive ROS content in the cell leads to overoxidation of the peroxidase cysteine (C_P_-SO_2_H/C_P_-SO_3_H) of Prdx6, which in turn leads to an increase of Ca^2+^-independent activity of phospholipase А2 (aiPLA2) [36]. The functional relation of these two Prdx6 activities was demonstrated in in vivo experiments. H_2_O_2_ was added to HeLa cell culture in different (1–1000 µM) concentrations, and arrest of cell cycle in G_2_-M phase was observed already at concentrations above 100 µM, which correlated with overoxidation of peroxidase cysteine of Prdx6 and elevation of aiPLA2 activity proportionally to H_2_O_2_ concentration [36]. Besides that, independently of the oxidation level of the peroxidase centre, Prdx6 phospholipase activity is increased by more than 10 times after specific phosphorylation of Thr177 residue by mitogen-activated protein kinases, MAPKs (ERK2, p38γ and p38δ) [120]. Activation of phospholipase aiPLA2 activity of Prdx6 has been shown to lead to growth of the level of lysophospholipids and fatty acids, which play a role of secondary messengers in both normal and pathological states [121]. The phospholipase activity of intracellular Prdx6 has been shown to stimulate signaling pathways (p38, PI3K/Akt) and facilitate arachidonic acid formation. Arachidonic acid, in turn, affects Src (SFK) kinases, which stimulate cell growth and division [89,122]. 

It has been shown that Prdx6 stimulates cell proliferation via JAK2/STAT3 signaling pathway [80]. Meanwhile, Prdx6 interacts directly with JAK2 protein, as immunohistochemical analysis showed colocalization of these proteins in lung cancer cells. Prdx6 affects the expression level of pro-inflammatory cytokines, especially CCL5 chemokine stimulating cell division, via JAK2/STAT3 signaling pathway [80].

Elevation of Prdx6 content in HeLa cells has been shown to provide resistance to TRAIL-(TNF-dependent apoptosis-inducing ligand)-induced apoptosis. Prdx6 binds to DED-domain (Death Effector Domain) of initiatory caspase-10 and thus prevents the formation of DISC (Death-Inducing Signaling Complex) and downstream activation of effectory caspases (caspase-3 and caspase-7) [85]. Besides that, in vitro studies testified that Prdx6 binding to caspase-10 decreases along with the growth of H_2_O_2_ amount added and, on the contrary, increases upon addition of dithiothreitol (DTT) as a reducing agent, speaking for the dependence of interaction of Prdx6 with caspase-10 DED-domain on the degree of reduction of the Prdx6 peroxidase center. Thus, both endogenous and exogenous Prdx6 can play an important anti-apoptotic role, blocking apoptosis progression via binding and inactivating key regulators of programmed cell death [85].

Moreover, endogenous Prdx6 has been shown to play an important role in suppression of mitochondrial ROS (mROS) generation. Upon activation of NF-kB mediated by TLR4 stimulation, TRAF6-ECSIT complex is induced, which facilitates OXPHOS-dependent mROS generation. Endogenous Prdx6 has been shown to bind to C-terminal domain of TRAF6 protein and prevent TRAF6-ECSIT complex formation and mROS generation [123].

Thus, the radioprotective potential of endogenous Prdx6 is related to both its catalytic properties and various signaling and regulatory roles of this protein in the cell.

### 5.2. Exogenous Prdx6

**Figure 11:**
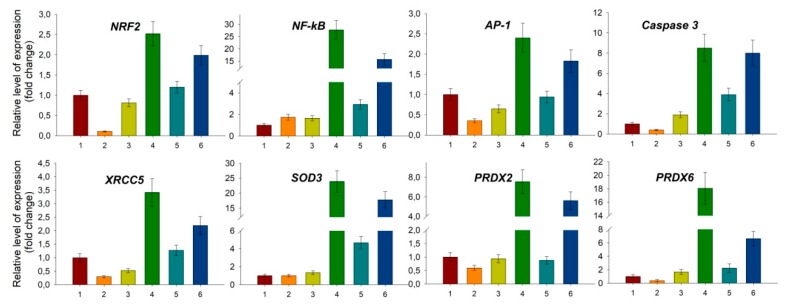
Changes in the expression level of some genes in bone marrow cells 24 hours after whole-body irradiation at a dose of 1.5 Gy and the effect of intravenous administration of Prdx6 and Prdx6-C47S (20 µg/g) 15min before irradiation. The data presented as mean ± SEM (*n* = 5). 1—intact mice; 2—non-irradiated mice received Prdx6 (20 µg/g); 3—non-irradiated mice received Prdx6-C47S (20 µg/g); 4—irradiated mice; 5—irradiated mice received Prdx6 (20 µg/g), 6—irradiated mice received Prdx6-C47S (20 µg/g).

As discussed above, exposure of a living organism to ionizing radiation leads to a burst of ROS level in the cells, which results in the development of oxidative stress. Injection of exogenous antioxidant enzyme Prdx6 before irradiation can affect the level of peroxides in the organism, thus preventing the development of oxidative stress and normalizing the redox status of the cells. A question arises: how can Prdx6 present in bloodstream neutralize ROS in the cells if it does not pass into them? It is known that, beside passive diffusion, hydroperoxides can be actively transported from cells to intercellular space by aquaporins [124,125,126]. Therefore, exogenous Prdx6 present in extracellular (intercellular) space can participate in elimination of both peroxides formed in intercellular space and peroxides excreted from the cells (Figure 10). As it was noted before, NRF2 is a key transcription factor regulating cell response on oxidative stress, which affects the expression of many antioxidant enzymes [46,47,127]. It was shown that Prdx6 injection in intact animals decreased NRF2 gene expression in a dose-dependent manner, and Prdx6 injection before 1.5 Gy irradiation of mice normalized NRF2 transcription level virtually to normal values (Figure 11), which is obviously related to a decrease in ROS level in the cells. Conversely, injection of Prdx6-C47S in intact animals and those irradiated at a dose of 1.5 Gy did not significantly affect the NRF2 expression level in animal bone marrow cells (Figure 11).

Another crucial transcription factor participating in response to ionizing radiation is NF-kB. NF-kB is known to play an important role in resistance of cells to radiation [128], and elevated ROS level, as in the case of NRF2, which significantly increases NF-kB gene expression [129]. Moreover, NF-kB and NRF2 signaling pathways are closely interconnected. An increase of NF-kB suppresses NRF2 gene expression and vice versa [130]. NRF2 activity suppression can be achieved also via competitive interaction of NF-kB with CBP (CREB binding protein), which is a transcription coactivator of NRF2, and via NF-kB-mediated activation of HDAC3 (histone deacetylase 3), which locally reduces histone acetylation in the ARE locus, thus hampering NRF2 binding to ARE and transcription of antioxidant response genes [131]. During inflammation reactions, a reverse situation can take place, when NRF2 inhibits NF-kB signaling pathway which involves RAC1 protein (small GTPase protein of Rho family) [132]. A relation between NF-kB and NRF2 has also been shown in Nrf2-null mice. The levels of NF-kB and pro-inflammatory cytokines controlled by this factor are significantly higher in these mice than in wild-type animals [133]. Thus, NF-kB and NRF2 pathways are closely related to each other. The common final goal of each of them is avoiding apoptosis and providing cell survival under stress conditions, but the common goal is achieved via different ways. The conditions of «switching» between NF-kB and NRF2 pathways essentially depend on biological species, tissue type, and physiological state of the organism [134,135], and the key role in this process can belong to peroxiredoxins.

Exogenous Prdx6 can affect NF-kB level in various ways: First, as in the case of NRF2, by regulating ROS level in the cell; second, via TLR4/NF-kB signaling pathway. It was shown that after one day following injection of exogenous recombinant Prdx6, the expression level of NF-kB in intact mice is slightly increased (by 2–3 times) (Figure 11). After one day following exposure of mice untreated with Prdx6 to ionizing radiation at 1.5 Gy, a drastic burst (of around 30 times) of the expression of expression level and some genes regulated by NF-kB (*SOD3*, *XRCC4*, *XRCC5*, *ATR*) took place in the animal bone marrow. It must also be noted that this effect on the expression of the studied genes was preserved up to 30 days after the exposure to ionizing radiation. The obtained data on NF-kB activation correlate with the results of work [136], where mice were exposed to a dose of 1 Gy and NF-kB activation took place in 3 h, 24 h, and 1 month after irradiation. Injection of Prdx6 before the exposure decreased the expression of NF-kB and genes regulated by this transcription factor to the values close to those in intact animals, which could possibly be related to ROS suppression (Figure 11).

Peroxiredoxins are localized predominantly inside the cell, except the secreted forms (Prdx4 and Prdx6), but after damage of plasma membrane by different factors (viral/bacterial infections, toxins, ionizing radiation, etc.) they appear in intercellular space and play a role of danger signals (DAMP—Damage-Associated Molecular Patterns) [137]. DAMPs can play both positive and negative roles: in some cases they activate the immune system and trigger regenerative processes, in other cases they stimulate angiogenesis and tumor growth [52,137,138,139,140,141]. The signaling function of exogenous Prdx6 is likely to be realized via the TLR4/NF-kB signaling pathway [142]. Quite recently, Prdx6 released during ischemia damage of the brain from broken cells was shown to play a role of an endogenous ligand of the TLR4 receptor [142]. Influence on the TLR4/NF-kB signaling pathway has been also shown for other members of the peroxiredoxin family [143]. Interaction of Prdx6 with TLR4 triggers a cascade of processes, the leading role being played by NF-kB (Figure 11), which allows launching emergency reparation processes and suppressing apoptosis development [144]. Besides that, we assume that intravenous injection of recombinant Prdx6 in animals 15 min before the exposure to X-rays can lead to a pre-conditioning effect. Up to the moment of action of ionizing radiation, cells have already launched the reparation mechanism and antioxidant response via the stimulation of the TLR4/NF-kB signaling pathway by injected exogenous Prdx6, which is recognized as DAMP. So, the last stimulus, action of ionizing radiation, does not lead to a synergistic increase of NF-kB expression. Meanwhile, intravenous injection of recombinant Prdx6, which turned out to be the most effective, allows exogenous Prdx6 to distribute around the organism in the fastest manner and to reach TLR4 receptors on cell surfaces in comparison to intramuscular and intraperitoneal injections. Thus, the activation of the NF-kB signaling pathway, which happens due to both binding of Prdx6 to TLR4 receptor and regulation of intracellular/extracellular ROS formed under the exposure to ionizing radiation, allows the cells to be protected from apoptosis by triggering inflammatory and reparation processes and activation of anti-apoptotic factors [144].

The anti-apoptotic effect of exogenous Prdx6 can be related to a transcription factor AP-1, which is one of the key regulators of apoptosis. Injection of exogenous Prdx6 suppresses the expression level of AP-1 and caspase-3 (Figure 11). It should be mentioned that the activation of AP-1 transcription factor is also mediated by ROS. Elevation of ROS content provides oxidation and activation of ASK-1 kinase (Apoptosis Signal regulating Kinase 1), which in turn activates protein kinase JNK (c-Jun N-terminal kinase). JNK activates AP-1 [145]. JNK/AP-1 signaling pathway, contrary to NF-kB and NRF2, stimulates apoptosis of the cells [146,147,148]. Inhibition of JNK/AP-1 pathway activation by antioxidants has been shown to prevent H_2_O_2_-induced apoptosis [149,150,151]. Being a powerful antioxidant, exogenous Prdx6 lowers ROS concentration in the cells and in this way, probably, prevents activation of ASK-1 and AP-1. Thus, apoptosis suppression in the presence of exogenous Prdx6 can be related to NF-kB activation and suppression of the JNK/AP-1 signaling pathway.

Besides that, recombinant Prdx6 has been recently shown to form ion-selective channels in artificial membranes in in vitro studies. Meanwhile, the oxidized form of Prdx6 is incorporated into the membrane much more effectively, than the reduced one, which may be explained by increased phospholipase A2 activity [152]. It is important to note that earlier phosphorylated Prdx6 (with more active aiPLA2) was shown to move to the plasma membrane in intact cells (mouse pulmonary microvascular endothelium and alveolar macrophages) [153]. Moreover, treatment of A549 cells with peroxides leads to lipid peroxidation and translocation of Prdx6 from the cytosol to the cell membrane [154]. So, Prdx6 could be incorporated into the cell membrane (through aiPLA2), thus performing a function of ion channel and playing an important regulatory role. 

Thus, we suppose that the basics of the radioprotective properties of Prdx6 are, on the one hand, its ability to neutralize a wide range of ROS, and, on the other hand, its signaling and regulatory activity, which facilitates triggering the cellular mechanisms of restoration of distorted redox homeostasis.

## 6. Practical Aspects of Prdx6 Radioprotective Action

### 6.1. Prdx6 Suppression in Treatment of Radioresistant Cancer

Cancer cells have elevated ROS level compared to normal ones, which is related to accumulation of internal metabolism distortions and effect of external factors such as hypoxia, increase of metabolic activity of oxidases, lipoxygenases, impairment of mitochondria functioning, effect of immune cells of the organism, etc. To be protected from oxidative stress, cancer cells developed a powerful system of antioxidant protection, its most important elements being peroxiredoxins. As mentioned before, high Prdxs level is observed in many cases of cancer disease, particularly in types resistant to ionizing radiation [94,155]. Application of small interfering RNAs (siRNAs) for *PRDXs* gene suppression in combination with chemotherapy could be an effective approach in cancer treatment. Delivery of siRNAs to the cell can be carried out in various ways; more can be found in the following reviews [156,157]. Particularly, this strategy was successfully applied in both in vitro and in vivo conditions when suppressing *PRDX1* expression in human lung cancer and colon cancer [90,158]. Suppression of *PRDX2* expression by siRNAs enhances the efficacy of the action of radiation and cisplatin on gastric cancer cells [159]. A combined use of chemotherapeutic agents producing ROS or ionizing radiation [160] with specific inhibitors of Prdxs seems also quite a promising approach. Such inhibitors are auranofin, adenantine, imexon, buthionine, sulfoximine, etc. For instance, SK053 compound has been shown to covalently bind and inhibit thioredoxins, which play a role of reducing agents in the catalytic cycle, and all peroxiredoxins, thus blocking cell division and activating apoptosis, in the Burkitt lymphoma model [161,162,163]. A specific inhibitor of Prdx6, thiacremonone discovered recently in garlic extract, is a dose-dependent suppressor of lung cancer cell growth (lines A549 and NCI-H460), which acts through triggering apoptosis [164]. SVT toxin from Turan blunt-nosed viper (*Vipera lebetinaturanica*) indirectly (affecting AP-1) inhibits *PRDX6* expression. Studies in animal models of lung cancer (A549 and NCI-H460) demonstrated effective dose-dependent alleviation of Prdx6 level and apoptosis stimulation by SVT [165]. Thus, specific suppression of Prdx6 can be a promising approach in the treatment of some radioresistant types of cancer.

### 6.2. Prdx6 Application as a Radioprotective Agent

Studies showed that Prdx6 injection prior to whole-body irradiation in sublethal and lethal doses significantly decreases the severity of radiation damage and increases the survival rate of animals. Assessment of Prdx6 as a radioprotector opens a new way for creating modern effective and safe radioprotectors. However, it should be noted that extensive clinical trials of Prdx6 as a drug are required. As noted before, there is no ideal radioprotector up to date. The most effective chemical radioprotectors have serious adverse effects, which restrict their application in effective concentrations. On the contrary, Prdx6 is a natural radioprotector and does not exert toxicity, while its efficiency is not lower than that of many known synthetic radioprotectors (Table 2). Prdx6-based preparations could be applied individually (perhaps in a complex of liposomes or perfluorocarbons) [112] or in combination with conventional modern radioprotectors from common practice, which could significantly decrease the probability of adverse effects and severity of the consequences of ionizing radiation exposure [166]. We suggest that the most promising is the application of radioprotective agents with different mechanisms of action, for example, Prdx6 as an antioxidant and indralin [97,167,168], an agent lowering oxygen consumption by the tissues, as a hypoxant, which could provide a synergistic radioprotective effect.

## 7. Conclusions

There is no doubt that Prdx6, an important antioxidant enzyme, plays a leading role in maintaining redox homeostasis in mammalian cells [19,169,170]. Moreover, being a signaling and regulatory protein, Prdx6 is involved in the regulation of many cellular processes [38]. Impairment of Prdx6 function in the cell, alteration (growth or reduction) of its expression level, and change of its localization lead to development of various pathologies [31,155,171].

Experiments in animal models of diseases accompanied by development of oxidative stress showed that application of exogenous Prdx6 can correct the pathologic process course and facilitate faster recovery of damaged organs and tissues [103,106,107,108]. Particularly, exogenous Prdx6 application alleviates the severity of ionizing radiation damage, increasing the survival rate of animals exposed to sublethal and lethal doses [110,111]. Thus, Prdx6 can be considered as a promising radioprotective agent for alleviating the pathological effect of ionizing radiation on mammalian organism.

## Figures and Tables

**Table 1:** Carcinogenic role of Prdx6 in various chemo- and radio-resistant forms of human cancer.

Type of Cancer	Mechanism of Action/Molecular Target	References
Brain	resistance to reactive oxygen species (ROS)	[77]
Lungs	resistance to ROS, activation of JAK2/STAT3, stimulation of metastasis, activation of iPLA2 activity, P38 activation via PI3K/Akt	[78,79,80]
Breast	resistance to ROS, stimulation of metastasis, stimulation of expression uPAR, Est-1, MMP-9, RhoC, TIMP-2	[81,82]
Esophagus	resistance to ROS	[83]
Stomach	resistance to ROS, stimulation of metastasis, suppression of caspase-8 activation	[84]
Cervix	resistance to ROS, suppression of TRAIL activated caspase-10	[85]
Liver	resistance to ROS	[86]
Ovaries	resistance to ROS, stimulation of metastasis	[87]
Bladder	resistance to ROS, stimulation of NF-kB	[51]
Prostate	resistance to ROS, stimulation of metastasis	[88]
Skin	resistance to ROS, stimulation of metastasis via activation of aiPLA2 activity	[89]

**Table 2:** The main classes of radioprotective compounds (mechanisms of action, time of administration (before or after irradiation), tissue specificity of action, and dose reduction factors).

Type of Radioprotective Compounds	Mechanism of Action	Time of Medication	Tissue Specificity	DRF *
Sulfhydryl compounds	- antiradical; - donation of H-atom; - formation of mixed disulfides; -hypoxia (↓); -redox regulation (↓)	before irradiation	all tissues	1.3–2.7
Antioxidants	- antiradical; - redox regulation (↓); - hypoxia (↓); -immunity stimulation (↓)	before irradiation	all tissues	1.1–1.3
Inhibitors of ACE	-effects on the renin–angiotensin system; - inhibition of collagen synthesis ? (↓)	after irradiation	does not protect the gastrointestinal tract	<1.2
Immunomodulators and cytokines	- stimulation of immunity; - cytokine production; - signaling cascades	before or after irradiation	mostly hematopoietic system	1.1–1.4
Prostaglandins	- tissue hormones - ? (↓)	before irradiation	hematopoietic system, gastrointestinal tract, hair follicles	<1.3
Metal salts and metallothionein	- induction of metallothionein; - antiradical	before irradiation	mostly hematopoietic system	<1.2
DNA_binding agents	- electron transfer; - compaction of chromatin (↓)	before irradiation	all tissues	<1.3
Hypoxia_inducing compounds	- hypoxia; - redox regulation (↓)	before irradiation	all tissues	1.2–1.5
Selenium containing compounds	- stimulation of glutathione peroxidase activity; - antiradical; - redox regulation (↓)	before or after irradiation	mostly gastrointestinal tract	<1.3
Nucleic acids and their derivatives	- antiradical; - effect on repair systems; - signaling cascades; - ? (↓)	before or after irradiation	all tissues	1.1–1.4
Fullerenes	- antiradical; - membrane protection; - ? (↓)	before exposure	all tissues	<1.3
Adsorbents	- binding of radionuclides; - acceleration of radionuclide excretion	after irradiation	gastrointestinal tract	**

*—dose reduction factor (DRF); **—DRF is difficult to estimate; since both radiation and chemical properties of the radionuclide contribute to mortality; ↓—The effect is weak or characteristic of selected members of the class only; ?—other mechanisms of action are possible.
